# Study on the mechanism of Wumei San in treating piglet diarrhea using network pharmacology and molecular docking

**DOI:** 10.3389/fvets.2023.1138684

**Published:** 2023-02-28

**Authors:** Huihui Yin, Wei Liu, Xiaoyu Ji, Guoqing Yan, Xueyan Zeng, Wu Zhao, Yanhua Wang

**Affiliations:** ^1^Guangxi Key Laboratory of Veterinary Biotechnology, Key Laboratory of China (Guangxi)-ASEAN Cross-Border Animal Disease Prevention and Control, Ministry of Agriculture and Rural Affairs of China, Guangxi Veterinary Research Institute, Nanning, China; ^2^Brain Function and Disease Laboratory, Shantou University Medical College, Shantou, Guangdong, China; ^3^Guangxi Mountain Comprehensive Technology Development Center, Nanning, China

**Keywords:** Wumei San, piglet diarrhea, traditional Chinese medicine systematic pharmacology, signal pathway, molecular docking

## Abstract

Wumei San (WMS) is a traditional Chinese medicine that has been widely applied in the treatment of piglet diarrhea (PD). However, the mechanism of WMS in PD has not been investigated. In this study, the main active compounds of WMS and the target proteins were obtained from the Traditional Chinese Medicine Systematic Pharmacology, PubChem, and SwissTargetPrediction databases. The molecular targets of PD were identified using GeneCards, OMIM, and NCBI databases. The common targets of WMS and PD were screened out and converted into UniProt gene symbols. PD-related target genes were constructed into a protein-protein interaction network, which was further analyzed by the STRING online database. Gene Ontology and the Kyoto Encyclopedia of Genes and Genomes enrichment analyses were performed to construct the component-target gene-disease network. Molecular docking was then used to examine the relationship between the core compounds and proteins. As a result, a total of 32 active compounds and 638 target genes of WMS were identified, and a WMS-compound-target network was successfully constructed. Through network pharmacology analysis, 14 core compounds in WMS that showed an effect on PD were identified. The targets revealed by GO and KEGG enrichment analysis were associated with the AGE-RAGE signaling pathway, PI3K-Akt signaling pathway, TNF signaling pathway, NOD-like receptor signaling pathway, IL-17 signaling pathway, and other pathways and physiological processes. Molecular docking analysis revealed that the active compounds in WMS spontaneously bind to their targets. The results indicated that WMS may regulate the local immune response and inflammatory factors mainly through the TNF signaling pathway, IL-17 signaling pathway, and other pathways. WMS is a promising treatment strategy for PD. This study provides new insights into the potential mechanism of WMS in PD.

## Introduction

Piglet diarrhea (PD) is a common disease, especially in 1- to 3-month-old piglets, with a high incidence in farming production (1). The incidence of diarrhea in weaned piglets is more than 30% and the mortality is 15%. PD may lead a decrease in the growth of piglets and a decrease in the feed reward. PD is also accompanied by physical decline, depressed immunity, weakened resistance to disease, and susceptibility to secondary and mixed infection of other infectious diseases. Without treatment, PD leads to a high death rate of piglets and severe economic injury to pig production (2, 3). PD is caused by general internal medicine diseases, sewage consumption, improper feed change, sudden weather changes, and cold stimulation. Other cases are caused by infectious diseases. Piglet yellow dysentery and piglet white dysentery are common bacterial diseases, and transmissible gastroenteritis, porcine epidemic diarrhea and rotavirus infection are common viral diseases in PD. The main strategies for treating PD in pig farms include antibiotics and medical levels of zinc oxide (4, 5). However, long-term medication and high zinc diet causes adverse effects on human health and the environment by contributing to the development of antimicrobial resistance among bacteria and to high soil concentrations of zinc, a heavy metal.

Because of their natural, eco-friendly, and safe nature, Chinese herbal feed additives and formulations have been used in increasing numbers in recent years for the prevention and treatment of PD (6, 7). According to the theory of traditional Chinese medicine, spleen and stomach weakness, cold dampness, dampness heat, and food injury are the important pathological factors of PD. The principle of treating PD is clearing heat and removing toxin, and astringing intestines and checking diarrhea. Wumei San (WMS) is a classic prescription for treating diarrhea in young animals. Its application history can be traced back to Hezi San from the earliest existing veterinary pharmacy *Anji Prescription* in Tang-Song period. WMS including *Mume Fructus* (MF), *Coptis chinensis* Franch. (CC), *Curcuma longa* L. (CL), *Terminalia chebula* Retz. (TC) and *Diospyros kaki* L.f. (DK) is now the latest recorded formula in *Chinese Veterinary Pharmacopeia* (2020 edition) II. Over thousands of years of evolution, only one or two herbs have changed in the prescription. MF, composed of organic acids, flavonoids and fatty acids, used to treat chronic cough, prolonged diarrhea, and other inflammation-related diseases (8). Previous studies have shown that weaned piglets fed with antibiotic-free diets supplemented with MF gained more weight and were healthier by modifying the gut microbial composition (9). CC is a Chinese herbal medicine with strong anti-inflammatory activity, and has obvious clinical medicinal value. Berberine, an isoquinoline alkaloid, mainly found in CC, with antibacterial effects on *Shigella* and *Escherichia coli*, is believed to exert gut health-promoting effects through modulation of the gut microbiota (10). CL has been used as a traditional Chinese medicinal material to treat gastrointestinal diseases for many years (11). TC is a widely used herbal drug in traditional medicine prescriptions. Chebulinic acid, a phenolic compound found in TC, is reported to exhibit both anti-inflammatory, anti-oxidant activity and anti-tumor property (12). DK is a popular cultivated and consumed fruits in China. Clinical studies showed that DK can help the gastrointestinal tract to digest and promote the recovery of appetite after diarrhea in sick pigs (13). The prescription of the compound WMS includes 15 g of MF, 24 g of DK, 6 g of CC, 6 g of CL, and 9 g of TC. The above five herbs are evenly combined, crushed, sieved, and combined. In this prescription, MF plays the role of promoting fluid production to quench thirst and astringing intestines to treat diarrhea as the main drug. TC and DK, as the auxiliary drugs, work through astringing intestines and consolidating. CL and CC are the assistant medicinal. CC takes effect of clearing heat and removing the toxin, and drying dampness to treat diarrhea. CL moves qi and activate blood to relieve pain. However, the mechanism of WMS is still unclear.

The multi-component, multi-target, and multi-channel characteristics of Chinese traditional veterinary medicine have made it difficult to elucidate the complex mechanisms, leading to a lack of data in pharmacological research. Network pharmacology has created a new framework for investigating how medications and disorders interact (14, 15).

**Figure 1:**
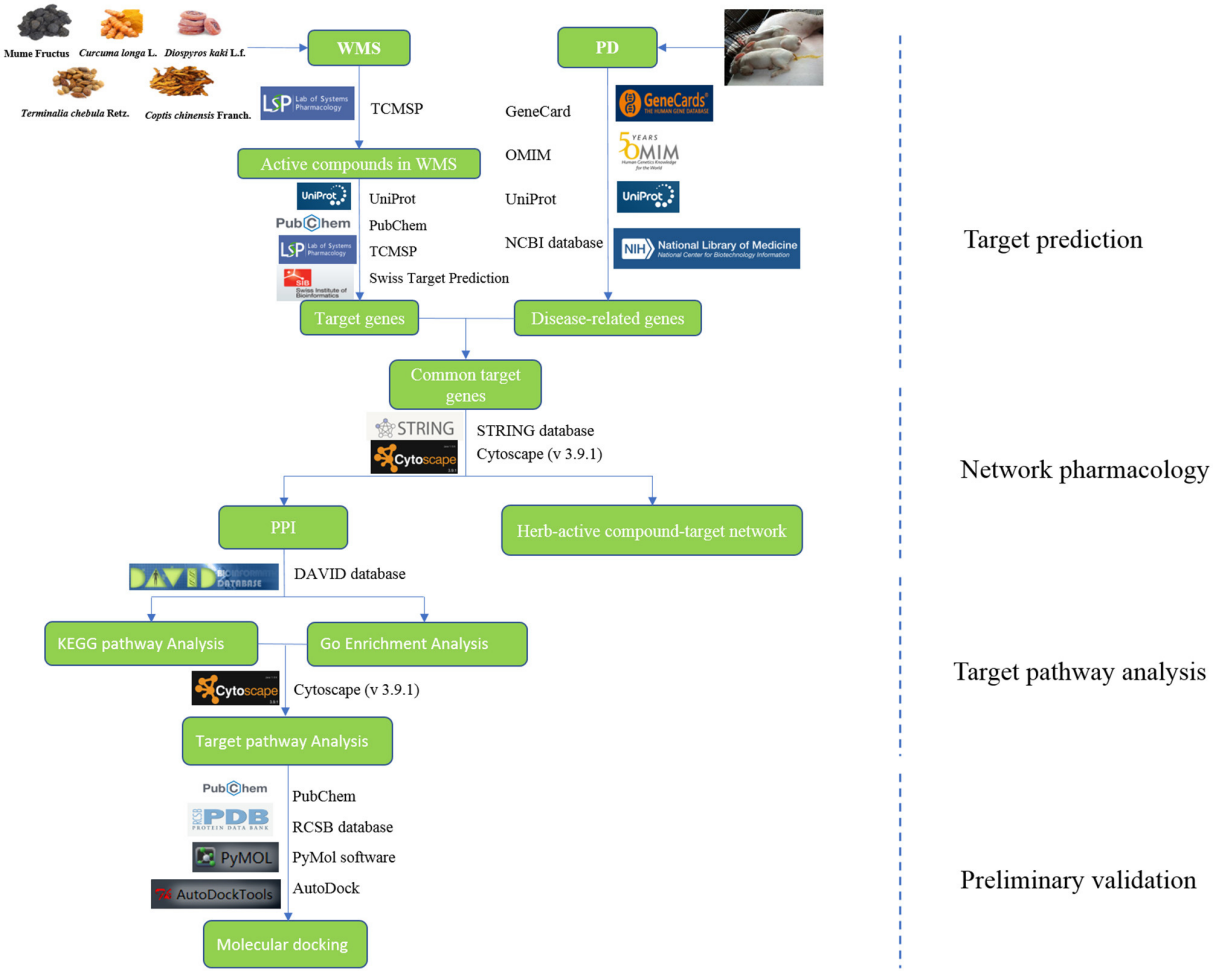
The flowchart of this study.

In this study, network pharmacology and molecular docking were applied to examine the potential mechanism of WMS in the treatment of PD. The study overview is shown in Figure 1.

## Methods

### Active components and target gene analysis of WMS

The formula of WMS containing MF, CC, CL, TC, and DK was obtained from *Chinese Veterinary Pharmacopeia* (2020 edition) II. The Traditional Chinese Medicine Systematic Pharmacology (TCMSP) (https://www.tcmspw.com/tcmsp.php) database, which is a systems pharmacology resource for traditional Chinese medicines or related compounds and offers details on the absorption, distribution, metabolism, and excretion properties of a drug with potential biological effects at a systematic level, was used to identify the compounds in the herbs of MF, CC, CL, TC, and DK. Oral bioavailability (OB) and drug likeness (DL) are two essential characteristics of medications taken orally. OB and DL can assess the effectiveness of a drug's systemic circulation, and the resemblance between a molecule and a known medication, respectively. The putative active components were screened using OB and DL in this research. Active substances were identified from prior investigations and data from key databases of Chinese herbal medicine. Active compounds were then screened and considered as putative main constituents and retained using OB ≥30 % and DL ≥0.18 based on data from prior studies and pertinent Chinese herbal medicine databases (16, 17). For example, an herb name was entered into the search box, and the compounds in the herb were examined. The potential active compounds were obtained then by setting OB ≥30 % and DL ≥0.18. The primary functional components for the treatment of PD that are not found in TCMSP were sorted out by examining previous studies (18, 19). Active compounds were input into the TCMSP database to obtain known targets. The structures of the active compounds were obtained from PubChem (https://pubchem.ncbi.nlm.nih.gov/) and imported into the SwissTargetPrediction database with relevant parameters for prediction of target genes (probability > 0). The potential targets were then collected. The target genes corresponding to the compounds were uniformly standardized in UniProt (http://www.uniprot.org/).

### Candidate targets of PD

“Piglet diarrhea” was used as the keyword to explore disease-related genes at GeneCards (https://www.genecards.org/), OMIM (https://www.omim.org/), and NCBI (https://www.ncbi.nlm.nih.gov/gene/). All potentially relevant genes were obtained. All the disease gene targets were then normalized and converted into gene symbols through UniProt database after removing the redundancy.

### Venn Diagram analysis

The predicted target genes of WMS and the projected target genes of PD were analyzed by Venn Diagram analysis (https://www.bioinformatics.com.cn/?keywords=%E6%96%87%E6%81%A9%E5%9B%BE).

### Construction of a network of herbs, natural compounds, and targets

From the above data, a network of herbs, active compounds, and common targets was constructed using Cytoscape (v 3.9.1), and the relationships among them were analyzed. Herbs, compounds, and targets were represented by nodes in the network, and their interactions were represented by edges connecting nodes.

### Construction of the protein—Protein interaction network

The potential target genes of WMS in PD were uploaded to STRING database (https://string-db.org/) (v 11.5) to draw a protein—Protein interaction (PPI) network. The organism was limited to “*Sus scrofa*.” The results showed the confidence of the interaction between the proteins by scores. The minimum required interaction score was selected at medium confidence data >0.4 to ensure the reliability of the analysis. The data from STRING database were then analyzed by the “Analysis Network” tool in Cytoscape 3.9.1 software to obtain a PPI network. The relevant parameters of degree (DC), betweenness centrality (BC), closeness centrality (CC), and stress were calculated for topology analysis on the PPI network to obtain the key targets.

### GO and KEGG pathway enrichment analyses

The potential targets from the intersection were imported into DAVID database (2021) (https://david.ncifcrf.gov/tools.jsp) for Gene Ontology (GO) and the Kyoto Encyclopedia of Genes and Genomes (KEGG) enrichment analysis with “*Sus scrofa*” as the species. *P* ≤ 0.01 was considered to be significantly enriched. The top 10 most significantly enriched GO biological processes (BP), cell component (CC), and molecular function (MF) and top 20 items of KEGG pathway results were then mapped (https://www.bioinformatics.com.cn/?keywords=pathway) to draw the enrichment bubble diagram.

### Construction of a network of herbs, compounds, pathways, and targets

The interaction information between intersection targets and the top 20 most significantly enriched KEGG pathways were combined with the screened drug components and intersection targets. Data were then uploaded into Cytoscape 3.9.1 to construct a network of herbs, active compounds, target, and pathway.

### Molecular docking

The 10 important compounds in accordance with the degree values in WMS were selected to dock to the top 10 core targets from the PPI analysis. The three-dimensional crystal structure of the target protein was downloaded from the RCSB database (https://www.rcsb.org/) and saved in pdb format after removing solvent and organic through the PyMol software. The 2D chemical structure of the compounds was obtained from PubChem and converted to mol2 format after minimizing energy through Chem3D 18.0. The ligand and receptor were then converted to pdbqt file format through AutoDockTools-1.5.7. Molecular docking was performed by AutoDock Vina 1.1.2. A binding energy of <-4.25 kcal/mol indicates that the ligand and receptor molecules bind spontaneously. The binding energy of <-5.0 kcal/mol indicates that ligand and receptor molecules are stably bound. The binding energy of <-7.0 kcal/mol indicates that the two have strong binding activity. Results with strong binding force were selected and visualized by Pymol software.

## Results

### The natural active ingredients in WMS

Using the databases, previous reports, and the criteria (OB ≥30%; DL ≥0.18) described above, the compounds of WMS were screened out. After the removal of non-target compounds, 8, 14, 3, 8, and 4 natural compounds of TC, CC, CL, MF, and DK were obtained, respectively. After removing any duplicate compounds, a total of 34 active compounds in the WMS formula were obtained (Table 1).

**Table 1:** Information of active ingredients in WMS.

**ID**	**Mol ID**	**Molecule name**	**OB (%)**	**DL**	**Source**
TC1	MOL001002	ellagic acid	43.06	0.43	TC
TC2	MOL002276	Sennoside E_qt	50.69	0.61	TC
TC3	MOL006376	7-dehydrosigmasterol	37.42	0.75	TC
TC4	MOL009135	ellipticine	30.82	0.28	TC
TC5	MOL009136	Peraksine	82.58	0.78	TC
TC6	MOL009137	(R)-(6-methoxy-4-quinolyl)-[(2R,4R,5S)-5-vinylquinuclidin-2-yl]methanol	55.88	0.4	TC
TC7	MOL009149	cheilanthifoline	46.51	0.72	TC
TC8	MOL006826	chebulic acid	72	0.32	TC
CC1	MOL000622	magnograndiolide	63.71	0.19	CC
CC2	MOL000762	palmidin A	35.36	0.65	CC
CC3	MOL000785	palmatine	64.6	0.65	CC
CC4	MOL001454	berberine	36.86	0.78	CC
CC5	MOL001458	coptisine	30.67	0.86	CC
CC6	MOL002668	worenine	45.83	0.87	CC
CC7	MOL002894	berberrubine	35.74	0.73	CC
CC8	MOL002897	epiberberine	43.09	0.78	CC
CC9	MOL002903	(R)-canadine	55.37	0.77	CC
CC10	MOL002904	berlambine	36.68	0.82	CC
CC11	MOL008647	moupinamide	86.71	0.26	CC
CC12	MOL013352	obacunone	43.29	0.77	CC
CC13	MOL002907	corchoroside A_qt	104.95	0.78	CC
A1	MOL000098	quercetin	46.43	0.28	CC, MF
CL1	MOL000493	campesterol	37.58	0.71	CL
B1	MOL000449	stigmasterol	43.83	0.76	CL, MF
B2	MOL000953	cholesterol	37.87	0.68	CL, MF
DK1	MOL000004	procyanidin B1	67.87	0.66	DK
DK2	MOL002773	β-carotene	37.18	0.58	DK
DK3	MOL000096	catechin	49.68	0.24	DK
DK4	MOL000073	epicatechin	48.96	0.24	DK
MF1	MOL000358	beta-sitosterol	36.91	0.75	MF
MF2	MOL000422	kaempferol	41.88	0.24	MF
MF3	MOL001040	(2R)-5,7-dihydroxy-2-(4-hydroxyphenyl) chroman-4-one	42.36	0.21	MF
MF4	MOL005043	campest-5-en-3beta-ol	37.58	0.71	MF
MF5	MOL008601	methyl arachidonate	46.9	0.23	MF

### Targets of the effective compounds of WMS

TCMSP, PubChem, and SwissTargetPrediction databases were used to predict the target genes of compounds in WMS. The results identified 638 targets, including 325, 447, 58, 75 and 271 targets for TC, CC, CL, DK and MF, respectively.

**Figure 2:**
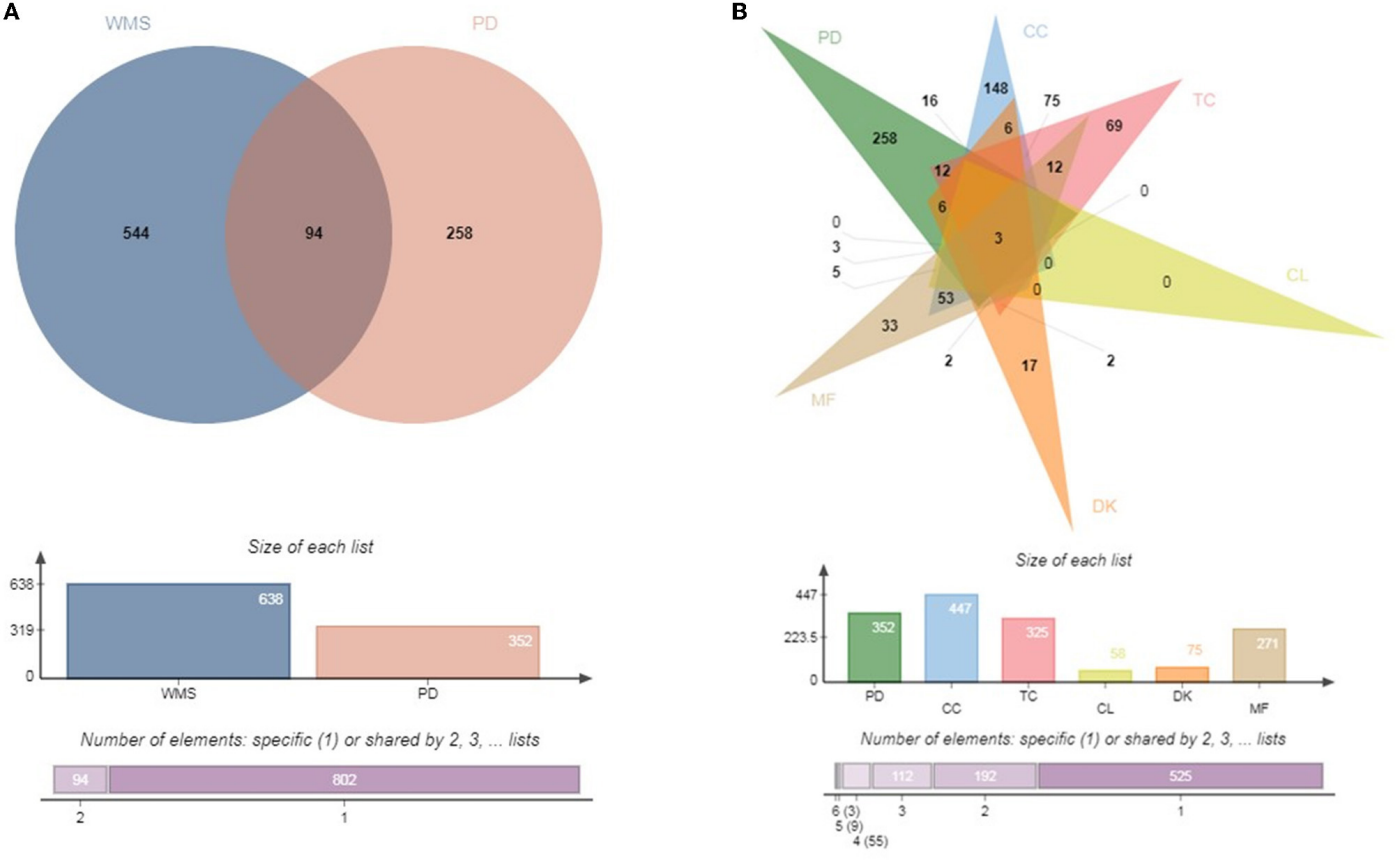
Venn diagram. **(A)** Common target genes of WMS and PD. **(B)** Common target genes of CC, TC, CL, MF, DK, and PD.

From the GeneCards website and OMIM, 352 genes were determined as highly likely to be associated with PD. The 352 candidate PD-associated genes were compared with the 638 target genes from WMS using Venn diagram analysis (Figure 2A). A total of 94 (11%) overlapping genes were extracted. The intersection between PD, CC, TC, CL, DK and MF which contains 3 common genes (Figure 2B).

### Construction of a network of herbs, natural compounds, and targets

**Figure 3:**
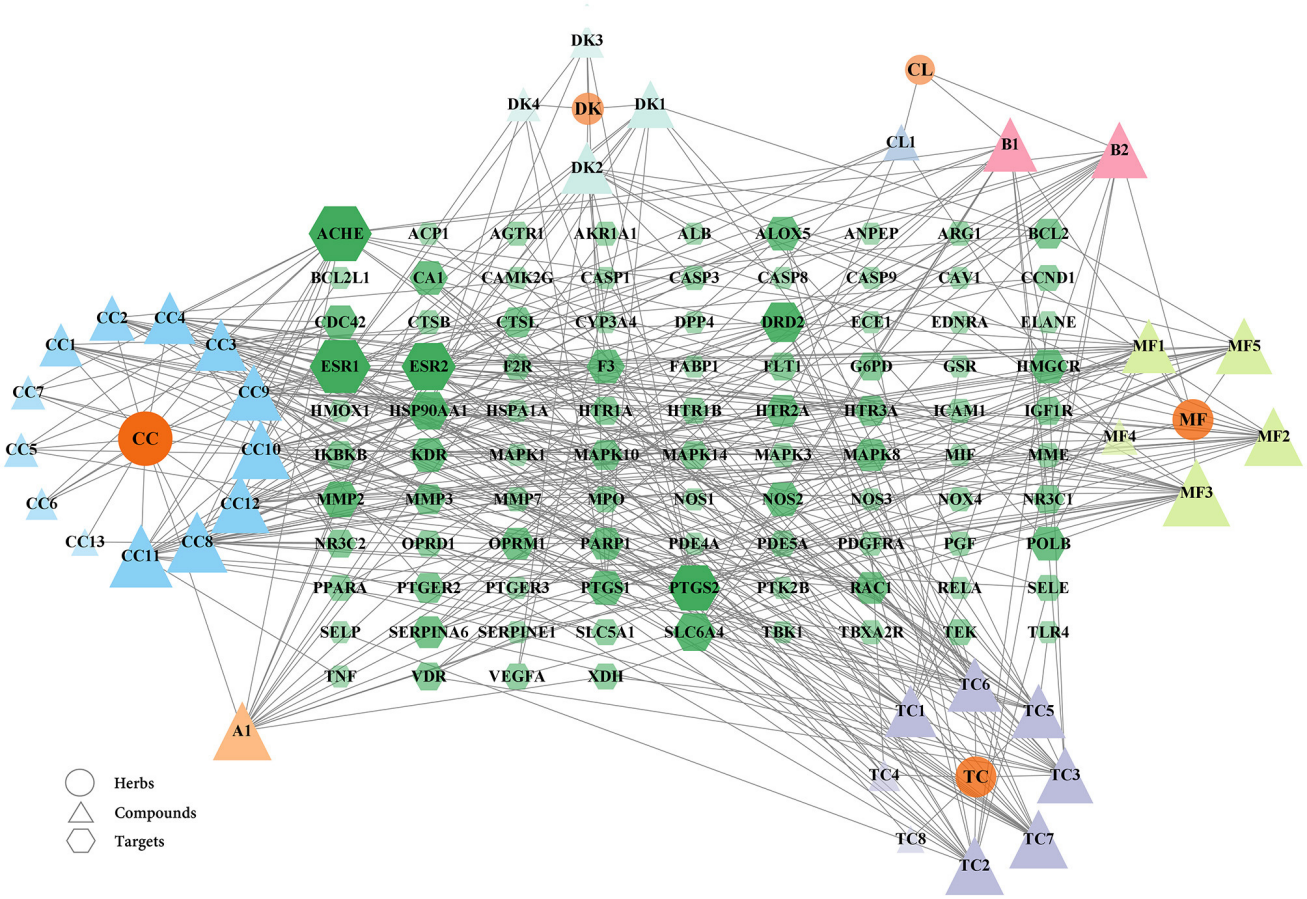
The herb-compound-disease target network. The ellipse nodes, the triangle nodes, and the hexagon nodes represent the herbs, compounds, and targets, respectively. The connections represent the interactions among the three. The node size and color are related to the degree value. The larger the node area and the darker the color in the figure, the more important it is in the network.

From the above data, a network of the complex interactions among components of WMS, effective compounds, and target genes was visualized using Cytoscape; the network comprised 133 nodes and 393 edges (Figure 3). The top 10 compounds by degree value are (2R)-5,7-dihydroxy-2-(4-hydroxyphenyl) chroman-4-one, moupinamide, epiberberine, sennoside E_qt, cheilanthifoline, quercetin, berlambine, obacunone, kaempferol, and 7-dehydrosigmasterol (Table 2). These compounds may be the core compounds of WMS responsible for anti-PD effects. This network allowed for easy observation of relationships among herbs, ingredients, and targets. These findings suggested that the pharmacological effects of WMS in the treatment of PD are the result of multi-component and multi-target effects.

**Table 2:** Topological parameters of active compounds.

**ID**	**Degree**	**Betweenness centrality**	**Closeness centrality**	**Stress**
MF3	20	0.111815254	0.403669725	32620
CC11	18	0.094447891	0.398791541	26248
CC8	17	0.060069500	0.378223496	18532
TC2	16	0.094894909	0.387096774	21988
TC7	16	0.048082513	0.389380531	25046
A1	16	0.051117664	0.389380531	19740
CC10	16	0.066485938	0.387096774	20366
CC12	16	0.069183574	0.391691395	20288
MF2	16	0.051702955	0.389380531	21782
TC3	15	0.060659147	0.378223496	21244

### Construction and analysis of the target PPI network

The 94 intersection genes were uploaded to the STRING database, and a PPI network was obtained depicting the BP of WMS treatment of PD *in vivo*. The network comprised 90 nodes and 607 edges. The results were then imported into Cytoscape to construct a network diagram.

**Figure 4:**
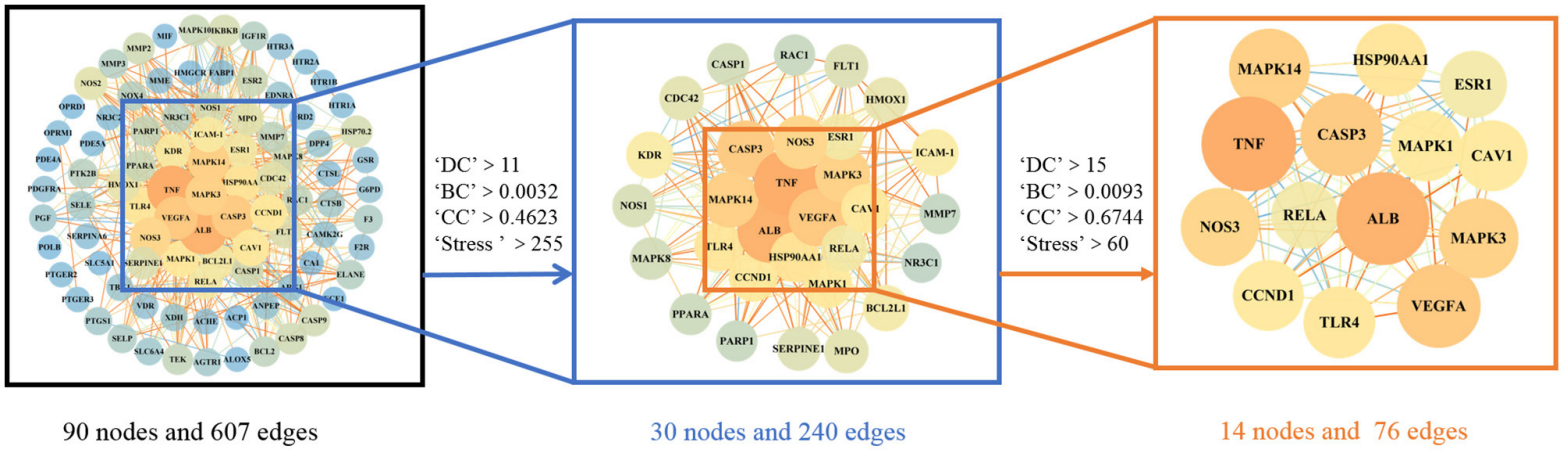
The topology screening process of the protein interaction network. The node size and color were related to the degree value. The color and size of the node are adjusted in accordance with the degree value. The darker colors and larger nodes indicate a larger degree value.

The PPI network and relevant parameters were obtained with the Analysis Network tool in Cytoscape software. Using the four parameters DC, BC, CC and Stress, the indicators above the median value were selected as the key indicators, and two screenings were performed. The critical values of the first screening were DC >11, CC >0.4623, BC >0.0032, and Stress >255. Through topological analysis, 30 key targets were obtained and screened again. The screening criteria were DC >15, CC >0.6744, BC >0.0093, and Stress >60. Finally, a total of 14 key targets of WMS acting on PD were obtained. The specific screening strategy is shown in Figure 4.

### GO and KEGG pathway enrichment analyses

After inputting common targets into the DAVID 2021 database, 145 KEGG pathways, 184 GO BP, 31 GO cell components and 57 GO molecular functions that met the enrichment criteria of *P* < 0.01 were identified.

**Figure 5:**
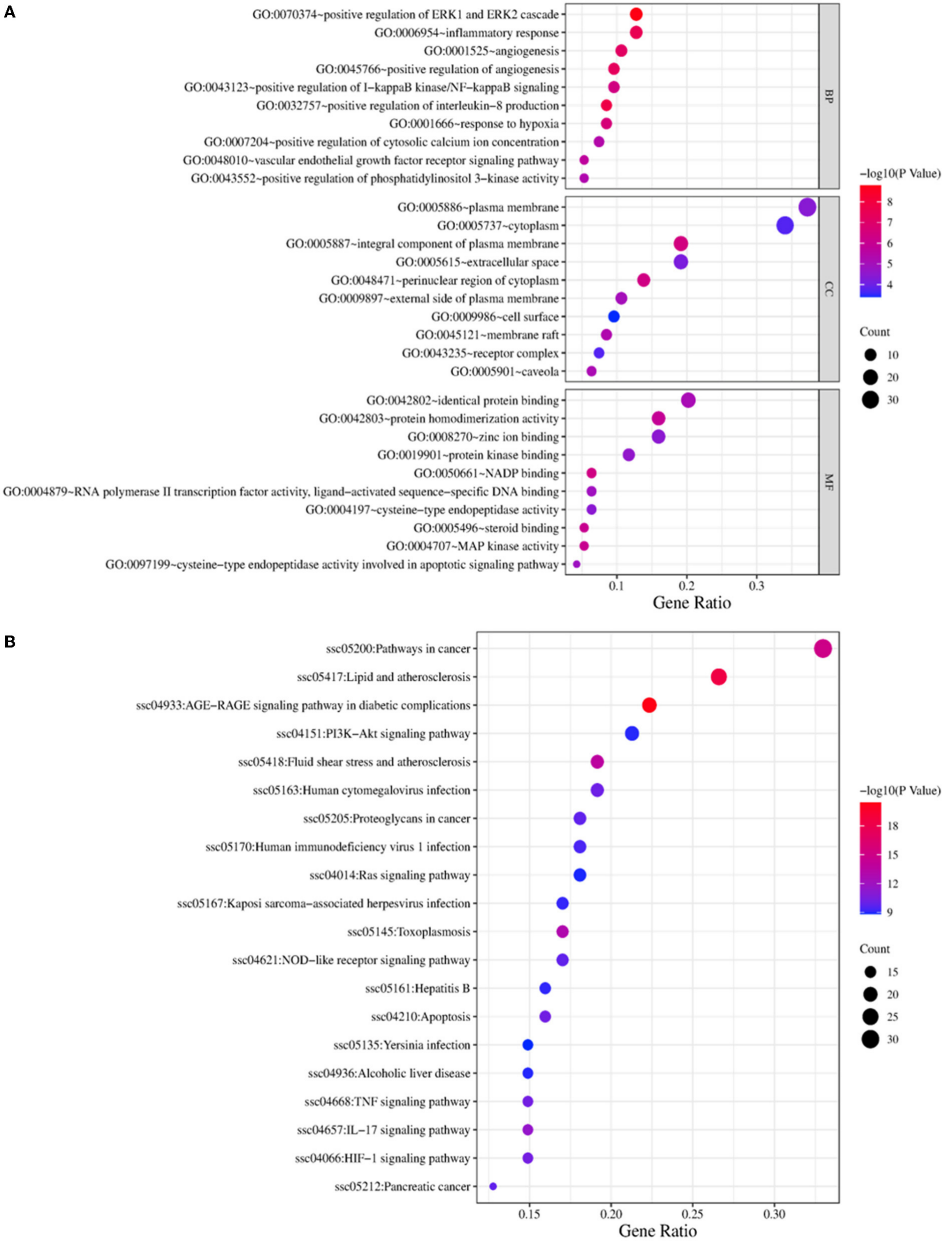
GO **(A)** and KEGG **(B)** pathway enrichment analysis.

The top 10 most significantly enriched GO BP were selected for analysis (Figure 5A). The major biological processes enriched were response to hypoxia, positive regulation of interleukin-8 production, inflammatory response, positive regulation of angiogenesis, and vascular endothelial growth factor receptor signaling pathway. The major molecular functions were MAP kinase activity, steroid binding, protein homodimerization activity, protein kinase binding, and protein homodimerization activity. The cellular components were mainly enriched in the perinuclear region of cytoplasm, caveola, extracellular space, receptor complex, and membrane.

The top 20 most significantly enriched KEGG pathways were selected for analysis (Figure 5B). The potential target genes of WMS in PD were involved in the AGE-RAGE signaling pathway, PI3K-Akt signaling pathway, TNF signaling pathway, NOD-like receptor signaling pathway, and IL-17 signaling pathway.

**Figure 6:**
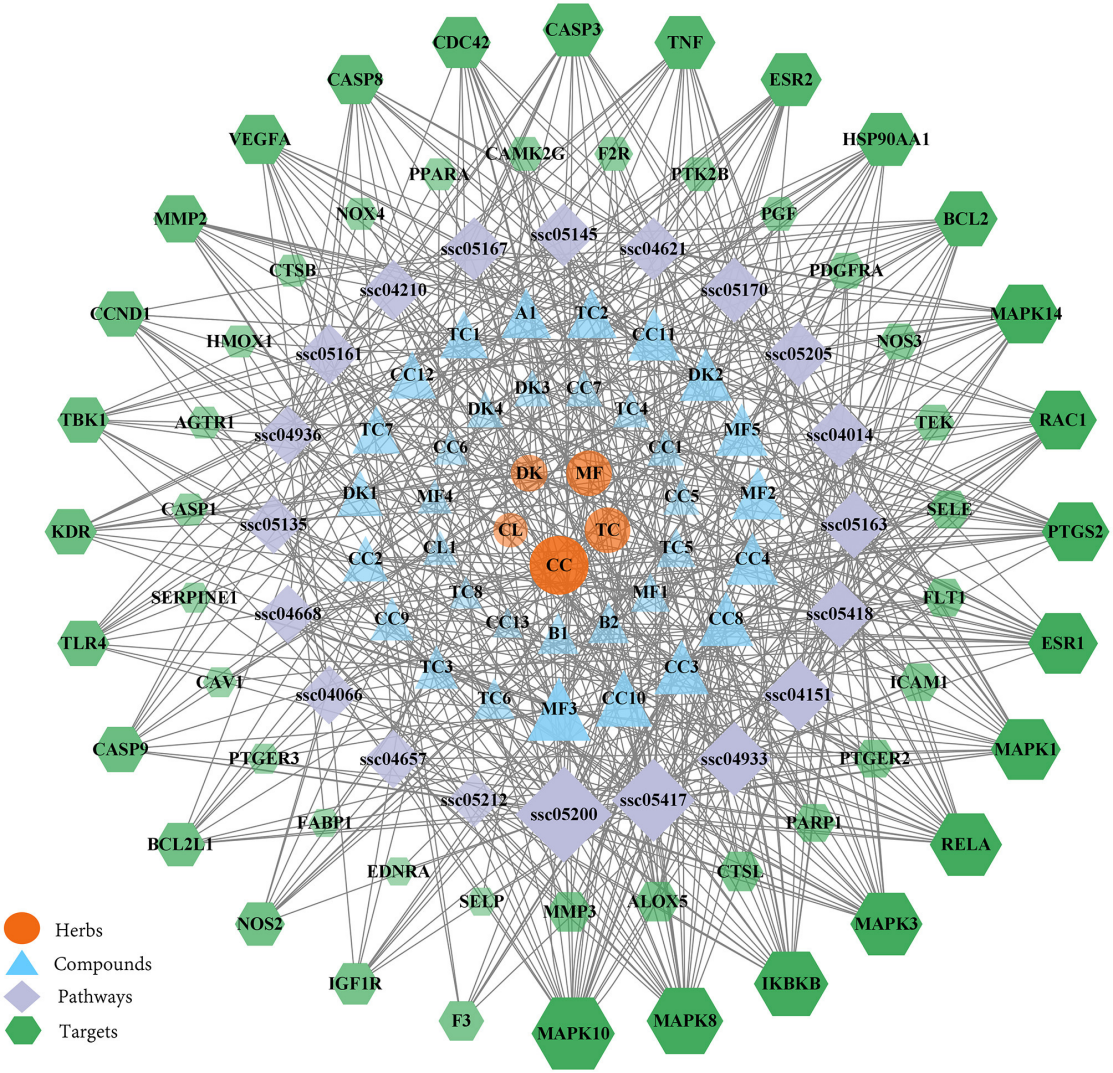
The herb-compound-disease target-pathway network. The orange ellipse, blue triangle, purple diamond, and green hexagon correspond to herbs, compounds, pathways, and target genes, respectively.

Using the herb, compound, target, and pathway analyses, an entire herb, chemical, target and pathway network was then constructed by Cytoscape using the top 20 signaling pathways. As shown in Figure 6, the interaction network has 114 nodes and 593 edges. The results showed that 55 of the 94 potential targets were involved in the top 20 pathways. These findings indicate that WMS has multi-component, multi-target, and multi-channel effects in the treatment of PD.

### Molecular docking

**Figure 7:**
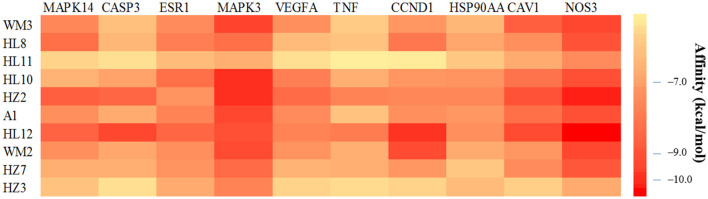
Molecular docking heat map. A darker color indicates a stronger binding force between the ligand molecule and the receptor protein.

From the degree analysis in the network, the 10 main active compounds (2R)-5,7-dihydroxy-2-(4-hydroxyphenyl)chroman-4-one (MF3), epiberberine (CC8), moupinamide (CC11), berlambine (CC10), sennoside E_qt (TC2), quercetin (A1), obacunone (CC12), kaempferol (MF2), cheilanthifoline (TC7), and 7-dehydrosigmasterol (TC3) were chosen for molecular docking verification with the 10 core targets MAPK14, CASP3, ESR1, MAPK3, VEGFA, TNF, CCND1, HSP90AA, CAV1, and NOS3. The statistical results of binding energy are shown in Figure 7.

**Figure 8:**
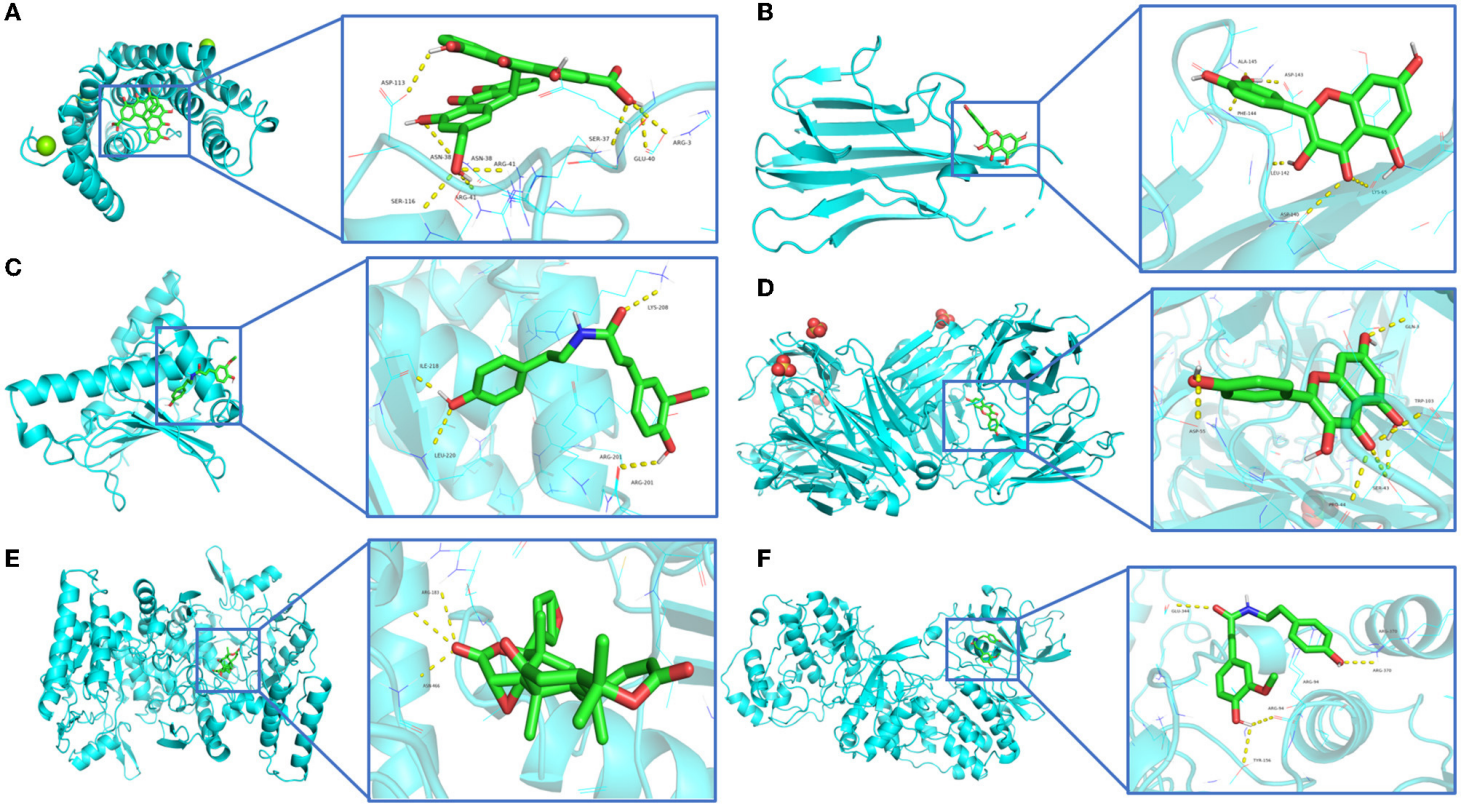
Molecular and key target docking verifications. **(A)** Sennoside E_q-ESR1, **(B)** Quercetin-TNF, **(C)** Moupinamide-HSP90AA1, **(D)** Kaempferol-CAV1, **(E)** Obacunone-NOS3, **(F)** Moupinamide-MAPK3.

Among the 100 receptor-ligand docking combinations, the binding energy of all molecules to proteins was <−5.0 kcal/mol, and 62 groups (62% of all combinations) had binding energy <−7.0 kcal/mol, which indicated that the main active ingredients in WMS have a strong binding activity with the core targets. The affinity of the compound obacunone to target NOS3 had the lowest binding energy of −11.1 kcal/mol. The binding modes of some key targets to the core active compounds are shown in Figure 8.

## Discussions

PD is a common disease in pig farms with a high morbidity and mortality that leads to productivity loss. Antibiotics and medical zinc oxide are the main treatment strategies for PD in pig production (20). However, long-term medication and a high zinc diet leads to adverse effects on human health and the environment by contributing to the development of antimicrobial resistance among bacteria and high zinc levels in soil. Therefore, the identification of a natural drug for PD prevention and treatment is critical. Some traditional Chinese medicines and formulations have been found to be effective in the treatment of PD. The WMS formula, which is composed of MF, CC, CL, DK, and TC, has the functions of regulating metabolism and enhancing immune effects. WMS has been clinically used in the treatment of PD in China and has a significant effect. However, the mechanism has been unclear.

In this study, network pharmacology was applied to analyze the effective components and mechanisms of WMS in the treatment of PD. A total of 32 active compounds in the WMS formula were obtained and 10 core compounds were identified. The compounds included terpenoids, flavonoids, alkaloids, and tannins. Flavonoids have the effects of reducing ulcerative colitis and anti-gastric ulcers, improving functional dyspepsia, inhibiting ileal motility, protecting gastric mucosa, and exerting antibacterial and antiviral effects (21). Quercetin is a flavonoid polyphenol molecule commonly found in vegetables, fruits, and Chinese herbal medicines; it promotes the reconstruction of epithelial tight junctions, enhances barrier integrity, and inhibits the production of proinflammatory cytokines such as IL17, TNF-α, and IL6 (22). Some of its metabolites reduce inflammation and prevent colitis (23). Previous studies showed that adding quercetin to the diet of weaned piglets increases the antioxidant capacity of piglets, regulates the structure and metabolism of intestinal microorganisms, and alleviates diarrhea and intestinal injury (24). Kaempferol is a polyphenol that is widely distributed in many vegetables, fruits, and beans. It has various pharmacological activities such as antiviral, apoptosis, anti-inflammation, and anti-oxidation effects (25). These effects may be related to kaempferol's inhibition of oxidative stress and attenuation of inflammatory factors, such as tumor necrosis factor-alpha (TNF-α), interleukin-6 (IL-6), cyclooxygenase-2 (COX-2), and nuclear factor κB (NF-κB) and the modulation of apoptosis and mitogen-activated protein kinase (MAPK) signaling pathways (26). Berberine is an alkaloid that reduces the expression of TNF-α, IL1β, and IL8 genes and the occurrence of intestinal inflammation through inhibiting the expression of TLR4 and NOD1 genes in intestinal mucosa (27, 28). Epiberberine is an isomer of berlambine; it exhibits anti-adipogenesis effects by modulating the Akt and ERK pathways, anti-dyslipidemia effects by inhibition on cholesterol synthesis, anti-cancer effects by impacting the p53/Bax apoptosis pathway, and antibacterial activities (29). 7-dehydrosigmasterol is a sterol compound. Phytosterols exhibit anti-inflammatory activities, improve the immunity of weaned piglets, and reduce the rate of PD (30). Epicatechin is a natural plant tannin compound that exerts anti-inflammatory effects by reducing the secretion of inflammatory cytokines and inhibiting the phosphorylation of P38 MAPK, extracellular signal-regulated kinase (ERK), and c-jun N-terminal kinase protein (JNK) in the MAPK signaling pathway (31). Supplementation of some tannins may help prevent PD in 21-day-old weaned piglets (32). These compounds form the material basis of the mechanism of action of WMS on PD, and further research is necessary to elucidate the detailed mechanisms.

The mapped PPI network was analyzed and 14 core targets were identified: MAPK14, HSP90AA1, TNF, NOS3, RELA, CCND1, TLR4, VEGFA, MAPK3, CAV1, MAPK1, ESR1, CASP3, and ALB. Intestinal inflammation is one of the main internal causes of piglet diarrhea, and the expression of the intestinal cytokines is one of the main characteristics of intestinal inflammatory response. Previous studies showed that, the gene expression of IL-6, TNF-α and IL-β in jejunum of piglets were increased (33, 34). The weaning stress may activate MAPK signaling pathways, NF-κB pathway and other pathways in the intestine (35, 36). Bacteria and lipopolysaccharide invade the intestinal mucosa of weaned piglets, activate the intestinal inflammatory signaling pathway, promote the transcription of downstream inflammatory factors, and cause intestinal inflammation in piglets. As can be seen from the Figure 3, the 14 core targets are the targets of the compounds in WMS that can directly act on PD. TLR4 corresponds to peraksine; MAPK14 corresponds to magnograndiolide, berberine, obacunone, and methyl arachidonate; VEGFA corresponds to (2R)-5,7-dihydroxy-2-(4-hydroxyphenyl)chroman-4-one, procyanidin B1 and β-carotene; TNF corresponds to obacunone; RELA corresponds to palmidin A; CAV1 corresponds to β-carotene. The results showed the characteristics of multi-component and multi-target effects of WMS in the treatment of PD. TNF is a critical cytokine with a wide range of sources. TNF-α is an indispensable immunomodulatory factor that maintains internal stability and functions in the resistance against various pathogenic factors. It has dual biological activities. At low concentrations, TNF-α is involved in resisting pathogenic microbial infection, promoting tissue repair, and regulating the inflammatory response (37). The initial line of defense against diarrhea is comprised of intestinal epithelial cells. TNF-α can induce epithelial cell apoptosis and destroys intestinal barrier function by rearranging the adhesion proteins in intestinal epithelial cells (38). The expression of NHE3 and DRA, the main transporter proteins regulating colonic Na^+^ inward transport and the main transporter protein regulating Cl^−^/HCO${}_{3}^{-}$ exchange in the apical membrane of mammalian intestinal epithelial cells, respectively, can be inhibited by the rising TNF-α expression in ulcerative colitis (39, 40). RELA is a member of the NF-κB family. Abnormal inflammation associated with inflammatory bowel disease is caused by excessive activation of RELA/NF-κB (41). MAPK14, also named P38α, plays an important role in the normal immune and inflammatory responses. The MAPK14 pathway plays a role in inflammatory bowel disease (42). TLR4 maintains immune tolerance and intestinal homeostasis, and inflammation is alleviated by TLR4 regulating the TLR4/NF-κB pathway (43, 44). CAV1 is a multifunctional protein, with roles in cellular defense by its inhibition of nitrosative stress and mucosal barrier injury (45). VEGFR is a receptor that binds to VEGF and initiates a signaling cascade that stimulates angiogenesis. A lack of VEGFR in newborn animals leads to intestinal microvascular dysplasia and colitis (46). Epidermal growth factor can promote the development of intestinal mucosal morphology in weaned piglets, activate gastric and intestinal digestive enzymes and disaccharidase, and improve weaning stress (47). We speculate that WMS exerts its effects on PD by acting on MAPK14, TNF, RELA, TLR4, VEGFA, CAV1, and other factors to regulate the inflammatory response, maintain the intestinal mucosal barrier, and improve stress.

GO enrichment analysis showed that WMS may play a therapeutic role by regulating the immune response of piglets, modulating cell growth, and regulating apoptosis. PD is caused by impaired intestinal barrier function, disruption of intestinal flora homeostasis, and disturbances in intestinal chemical, mechanical, and immune barriers (48, 49). The impaired intestinal barriers may promote bacterial translocation and the entry of allergic compounds from the intestine into the body, leading to increased immune response and susceptibility. GO enrichment analysis showed that the active ingredients of WMS exert activities on cell components and structures such as the cytoplasm, caveola, cell surface, extracellular space, receptor complex, and membrane, which are related to the maintenance of intestinal epithelial tissue integrity. Thus, WMS may prevent intestinal barrier damage and the entry of sensitive substances such as bacteria from the intestine into the body.

PD progresses through a variety of pathophysiological processes, including stimulation of intestinal structural damage, digestive dysfunction, oxidative stress, and inflammation response. KEGG pathway enrichment analysis showed that the pharmacological effects of WMS in treating PD included effects on the AGE-RAGE signaling pathway, PI3K-Akt signaling pathway, TNF signaling pathway, NOD-like receptor signaling pathway, and IL-17 signaling pathway. As can be seen from the Figure 6, the compounds berberine, moupinamide, palmidin A, obacunone, β-carotene, catechin, and methyl arachidonate in WMS modulate the AGE-RAGE signaling pathway by regulating MAPK14, TNF, VEGFA, RELA and other targets to treat piglets. Peraksine, β-carotene, catechin, and (2R)-5,7-dihydroxy-2-(4-hydroxyphenyl)chroman-4-one modulate the PI3K-Akt signaling pathway by regulating the TLR4, VEGFA, and other targets. Berberine, ellagic acid, sennoside E_qt, quercetin, chebulic acid, β-carotene, catechin, and epicatechin modulate the TNF signaling pathway, IL-17 signaling pathway and NOD-like receptor signaling pathway by regulating TNF, MAPK14, RELA, MMP3, CASP8, PTGS2, and other targets to treat PD. The AGE-RAGE signaling pathway elicits activation of multiple intracellular signal pathways involving NADPH oxidase, protein kinase C, and MAPKs, then resulting in NF-κB activity. NF-κB promotes the expression of pro-inflammatory cytokines such as IL-1, IL-6, and TNF-α (50). TNF induces a wide range of intracellular signal pathways including apoptosis, cell survival, inflammation, and immunity pathways. TNF-α is an indispensable immunomodulatory factor and an important downstream factor of the NF-κB pathway. When the intestinal epithelial cells was attacked by transmissible gastroenteritis virus, adding leucine can inhibit the expression of the pro-inflammatory factor TNF-α and anti-inflammatory factor IL10 by inhibiting the phosphorylation level of NF-κB, thus reducing the inflammatory response in the small intestine (51). The PI3K-Akt signaling pathway is activated by many types of cellular stimuli or toxic insults and regulates fundamental cellular functions such as transcription, translation, proliferation, growth, and survival. Previous studies showed that flavonoids ameliorate dysregulated inflammatory responses, the intestinal barrier, and gut microbiome in ulcerative colitis *via* the PI3K-AKT pathway (52). IL17 is associated with bacterial infection, and γδT cells secreting IL17 are the key component of the mucosal defense against infection (53). NOD-like receptors are representatives of mediating inflammatory responses. After activation, they can induce the release of inflammatory factors by mediating the NF-κB pathway and MAPK pathway (54). Together these results indicate that the AGE-RAGE signaling pathway, PI3K-Akt signaling pathway, TNF signaling pathway, IL-17 signaling pathway, and other pathways may play key roles in the effects of WMS on PD.

## Conclusion

WMS is a traditional Chinese medicine formula that is effective in treating PD. In this study, network pharmacology combined with molecular docking was applied to explore the mechanism underlying the effect of WMS on PD. The results indicated that WMS may exert its anti-PD activities through the TNF signaling pathway and IL-17 signaling pathway. The AGE-RAGE signaling pathway, PI3K-Akt signaling pathway, and NOD-like receptor signaling pathway may also be involved. Further experiments are required to elucidate the precise mechanism by which WMS functions in PD.

## Data availability statement

The original contributions presented in the study are included in the article/supplementary material, further inquiries can be directed to the corresponding author.

## Author contributions

HHY wrote the manuscript. HHY, WL, XYJ, and GQY designed the figures and edited the manuscript. HHY supervised data analysis and manuscript editing, in cooperation with XYZ, WZ, and YHW. All authors contributed to the article and approved the submitted version.
